# Perception of Time in Articulated Visual Events

**DOI:** 10.3389/fpsyg.2012.00564

**Published:** 2012-12-17

**Authors:** Gijs Plomp, Cees van Leeuwen, Sergei Gepshtein

**Affiliations:** ^1^Functional Brain Mapping Laboratory, Department of Fundamental Neuroscience, University of GenevaGeneva, Switzerland; ^2^Laboratory for Perceptual Dynamics, Katholieke Universiteit LeuvenLeuven, Belgium; ^3^Systems Neurobiology Laboratories, Salk Institute for Biological StudiesLa Jolla, CA, USA

**Keywords:** time perception, duration estimation, dilation, measurement, vision, uncertainty principle, optimality

## Abstract

Perceived duration of a sensory event often exceeds its actual duration. This phenomenon is called time dilation. The distortion may occur because sensory systems are optimized for perception within their respective modalities and not for perception of time. We investigated how the dilation of visual events depends on the duration and content of events. Observers compared the durations of two successive visual stimuli while the luminance of one of the stimuli was modulated at different temporal frequencies. Time dilation correlated with the frequency of modulation and the duration of the stimulus: the faster the modulation and the longer the stimulus duration, the larger the dilation. Notably, time dilation was also accompanied by a decreased sensitivity to stimulus duration. We show that these results are consistent with the notion that stimulus duration is estimated using measurement intervals of the lengths that depend on stimulus frequency content. Estimation of temporal frequency content is more precise using longer measurement intervals, whereas estimation of temporal location is more precise using shorter ones. As a result, visual perception will benefit from using longer intervals when the stimulus is modulated so that its frequency content is measured more precisely. A side effect of using longer temporal intervals is a larger uncertainty about the timing of stimulus offset (temporal location), ensuing time dilation and the reduction of sensitivity to duration. Our findings support the view that time dilation follows from basic principles of measurement and from the notion that visual systems are optimized for visual perception rather than for perception of time.

## Introduction

Visual systems have intrinsic temporal characteristics, but the perception of time and duration is hardly a natural visual task. Visual systems are likely to be optimized for measuring properties of visual stimuli, such as their location and content. It is therefore not surprising that the apparent duration of visual events depends on their visual content (Allan, 1979; Fraisse, 1984). For example, filled temporal intervals appear to last longer than empty ones (Goldfarb and Goldstone, 1963), and perceived duration increases with stimulus complexity (Schiffman and Bobko, 1974), stimulus size (Xuan et al., 2007), and temporal modulation of the stimulus (Brown, 1931; Roelofs and Zeeman, 1951; Goldstone and Lhamon, 1974; Lhamon and Goldstone, 1974; Kanai et al., 2006). This phenomenon depends on attention (Mattes and Ulrich, 1998; Tse et al., 2004) and reflects the perceived rather than the physical aspects of the event (Ono and Kawahara, 2007; Yamamoto and Miura, 2012).

Overestimation of duration is called *time dilation*. To explain this phenomenon, previous studies have posited that estimation of duration depends on memory. For example, perceived duration was proposed to be a function of the number of perceived changes in the event (Fraisse, 1963, 1984) or a function of stimulus complexity (Ornstein, 1969). In this view, the more complex stimuli appear to last longer because their perception requires a greater effort and a larger storage size than simpler stimuli. Of the temporal characteristics of visual stimuli, temporal frequency of luminance modulation was found to be the most important predictor of time dilation in dynamic visual displays (Kanai et al., 2006).

Here we explore the possibility that distortions of perceived duration can arise as side effects of the specialization of visual systems for estimation of visual parameters of stimuli, such as their locations and spatial and temporal frequency content. Outcomes of this estimation depend on the size of the interval of measurement. According to a well-established property of measurement (Gabor, 1946; Gepshtein et al., 2007), estimation of the frequency content of a signal is more reliable (more precise) over large intervals. And estimation of location is more reliable over small intervals. Visual systems would therefore benefit from engaging mechanisms characterized by increasingly large measurement intervals when stimuli are characterized by increasingly rich frequency content, impeding estimation of signal location.

In two experiments, we investigate how errors of duration estimation depend on visual properties of stimuli in a duration discrimination task. We present observers with two sequential stimuli while the luminance of one of the stimuli is subjected to temporal modulation at different temporal frequencies. We measure consequences of this modulation for perceived stimulus duration and response confidence, while controlling for known effects of stimulus order (Fechner, 1860; Hellstrom, 1985). We find that in addition to time dilation, the temporal modulation reduces the sensitivity to stimulus duration.

Using a numerical model of duration estimation, we show that these findings are consistent with basic principles of measurement. We assume that durations of visual stimuli are measured with variable temporal intervals, and that more strongly modulated stimuli activate mechanisms with longer measurement intervals. We show that longer measurement intervals lead to time dilation and *also* make the estimates of duration less precise, consistent with our finding of reduced sensitivity to stimulus duration of modulated stimuli. Taken together, these results support the view that temporal modulation of visual events engages visual mechanisms that use longer temporal integration intervals.

## Experiment 1

Using a two-interval forced-choice procedure, we studied how luminance modulation of visual stimuli affects their perceived durations.

### Materials and methods

#### Observers

Nine observers (mean age 22; six females) gave informed consent before the experiment. Participants provided written informed consent. All procedures were approved by the RIKEN ethics committee.

#### Apparatus and stimuli

The stimuli were gray disks presented in a darkened room on a 17-inch CRT screen with 100 Hz refresh rate using the open source Vision Egg software (Straw, 2008). Each disk had a diameter of 2° of visual angle. The viewing distance was 90 cm. Stimulus disks were presented sequentially on a black background at screen center.

One of the disks was the “standard” stimulus (of one of three fixed durations) and the other one was the “comparison” stimulus (of variable duration). Luminance of the standard stimulus was modulated: it was a sinusoidal function of time with the peak and through contrasts of 25 and 75% (1 and 25 cd/m^2^). The frequency of modulation was 3 or 7 Hz. The phase of modulation was randomized on every trial such as to avoid an association of luminance extremes (peaks and troughs of the waveform) with the onset and offset of the stimulus. The comparison stimulus had a constant luminance of 9 cd/m^2^.

#### Procedure

At the beginning of every trial, observers fixated a small central cross that subtended 0.5° of visual angle. Duration of fixation was sampled from a uniform distribution on the interval of 1000–1500 ms. Fixation was then replaced by the first stimulus (S1), followed by a blank inter-stimulus interval (ISI) whose duration was sampled from a uniform distribution on the interval of 500–1000 ms. After the second stimulus (S2) was presented, observers indicated with a key press whether S1 or S2 appeared to last longer.

On every trial, one standard stimulus (of fixed duration: 500, 800, or 1100 ms) and one comparison stimulus (of variable duration) were presented in a random order. The duration of the comparison stimulus was controlled by the adaptive psychophysical procedure QUEST (Watson and Pelli, 1983), as implemented in the Vision Egg package (Straw, 2008).

Experiment 1 consisted of four blocks of trials: one baseline block (with no stimulus modulation) and three blocks that included modulated stimuli. Within a block, each unique condition was repeated 30 times, in a random order. The baseline block contained all three standard stimulus durations without luminance modulation. The three standard durations were randomly interleaved and the presentation order of the standard stimulus was random (S1 or S2). The articulated blocks contained trials with modulated standard stimuli. One standard duration (500, 800, or 1100 ms) was used within a block, while factors Modulation Order (S1 or S2 modulated) and Modulation Frequency (3 or 7 Hz) were counterbalanced, resulting in four unique conditions.

### Results

We fitted cumulative normal distributions to the fractions of responses “comparison duration longer” for every modulation frequency, stimulus order, standard duration, and observer. In every case we determined the Point of Subjective Equality (PSE): the duration of the comparison stimulus for which the standard and comparison stimuli were equally likely to be reported as the longer stimulus. We operationally defined dilation as the difference between the PSE in the presence of modulation and the PSE in the absence of modulation (The latter was the baseline condition).

We evaluated how time dilation depended on factors Standard Duration (500, 800, or 1100 ms), Modulation Order (S1 or S2), and Modulation Frequency (3 or 7 Hz) using linear mixed-effects models (Pinheiro and Bates, 2000). Factor Observer was modeled as a random effect and factor Day was modeled as a random effect within Observer. The fixed effects and their interactions were assessed by ANOVA using the software environment R (R-Development-Core-Team, 2011).

**Figure 1:**
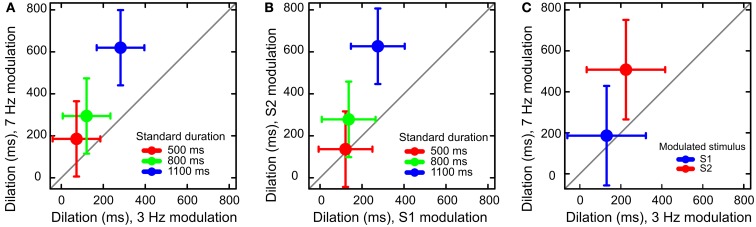
**Results of Experiment 1**. **(A)** Dilation increased as a function of both modulation frequency and standard duration. **(B)** Dilation increased more when S2 (the second stimulus) was modulated than when S1 was modulated: a manifestation of the time-order effect. **(C)** The effect of modulation frequency was larger for the higher frequency of modulation. Error bars represent 95% confidence intervals around the mean.

We found three main effects. Dilation increased with Standard Duration, *F*(1, 297) = 18.59, *p* < 0.001, with Modulation Order, *F*(1, 297) = 22.02, *p* < 0.001, and with Modulation Frequency, *F*(1, 297) = 29.81, *p* < 0.001. We observed a marginal interaction between Modulation Frequency and Standard Duration, *F*(1, 297) = 2.58, *p* = 0.07 (Figure 1A), suggesting that the increase of dilation across Standard Duration tended to be larger for 7 Hz modulation than 3 Hz modulation. There was a significant interaction of Modulation Order and Standard Duration, *F*(1, 297) = 6.65, *p* < 0.01. The latter interaction indicates that the increase of dilation across levels of Standard Duration was larger when S2 was modulated than when S1 was modulated (Figure 1B). Modulation Order and Modulation Frequency interacted too: *F*(1, 297) = 8.44, *p* < 0.01 (Figure 1C). The difference in dilation between modulation frequencies was largest when S2 was modulated.

Average slopes of the psychometric functions for all conditions are summarized in Table 1. The slopes increased with modulation and standard duration. But since we cannot dissociate sensitivity to stimulus duration from observers decision biases in this experiment, we relegate the analysis of sensitivity to Experiment 2.

**Table 1:** **Average slopes of psychometric functions measured in Experiment 1**.

	0 Hz	3 Hz	7 Hz
500 ms	76.29	76.62	108.73
800 ms	117.69	132.01	176.86
1100 ms	184.46	216.40	263.04

To summarize, we found that stimulus modulation led to overestimation of stimulus duration. This dilation effect was stronger for the modulation of 7 Hz than 3 Hz, for the stimulus that came later in the sequence of two stimuli, and it increased with stimulus duration.

## Experiment 2

We reasoned that temporal estimation of visual events is mediated by mechanisms optimized for visual perception. Visual perception of stimuli that contain temporal modulation of luminance is likely to rely more on mechanisms with long than short temporal integration intervals, because longer intervals are more suitable for estimation of stimulus frequency content (Gabor, 1946). If time dilation was caused by integration of stimuli over longer intervals, then the dilation is expected to be accompanied by reduced sensitivity to stimulus duration (We develop this argument in greater detail in the Discussion). We tested this hypothesis in Experiment 2 by separating the effects of observer sensitivity and response bias using the Receiver Operating Characteristic (ROC) analysis. In contrast to Experiment 1, now we used the method of constant stimuli and a rating procedure.

### Materials and methods

#### Observers

Six observers (mean age 22, four females) participated after giving informed consent.

#### Stimuli

The stimuli were as in Experiment 1. We used two standard durations (500, 1100 ms) and two modulation frequencies (0, 7 Hz). When modulation was present, only the standard stimulus was modulated (as in Experiment 1). Comparison durations were fixed at 0.25, 0.50, 0.75, 1.25, 1.50, and 1.75 times the standard duration.

#### Procedure

Observers responded which stimulus lasted longer using a six-point confidence rating scale with categories “certainly the first,” “probably the first,” “guessing the first,” “guessing the second,” “probably the second,” and “certainly the second.” Factors Standard Duration (500, 1100 ms) and Modulation Frequency (0, 7 Hz) were blocked in a fully crossed factorial design so that each block contained trials of one unique combination of the two factors. The four blocks were presented in a different random order across observers. Within every block, factor Standard Order (S1, S2) was randomized. Each unique condition was repeated 15 times per block for each of the six comparison durations. Three observers performed the four blocks twice, the other three observers once.

### Results

#### Psychometric functions

**Figure 2:**
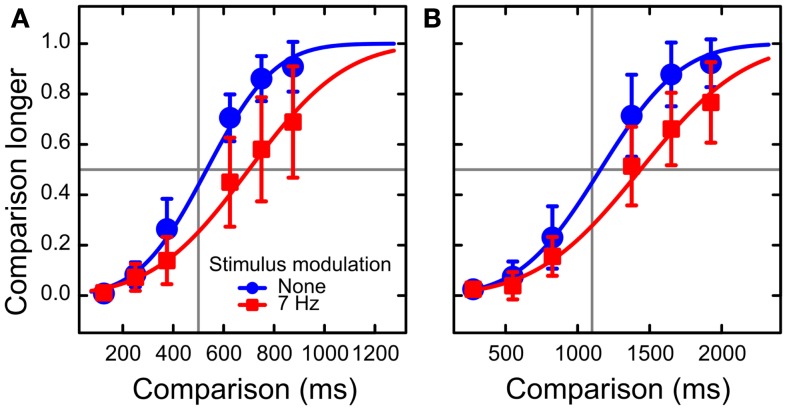
**Results of Experiment 2: psychometric functions**. The data are plotted separately for standard durations of 500 ms **(A)** and 1100 ms **(B)**. The red squares and blue circles represent the conditions of luminance modulation present and absent, respectively. The data are averaged across observers. The curves are the cumulative normal fits to fractions of reports that the comparison stimuli appeared to be longer than the standard stimuli. The psychometric functions for 7 Hz modulation are shifted to the right relative to those with modulation absent, which is a manifestation of time dilation. Error bars represent 95% confidence intervals around the means.

We fitted cumulative normal distributions to the fractions of responses “comparison duration longer” separately for each condition (Figure 2). Time dilation was on average 146 and 249 ms for the modulated standard durations of 500 and 1100 ms, respectively.

As in Experiment 1, we evaluated how time dilation depended on the factors of Standard Duration and Standard Order. The main effect of Standard Order was marginally significant, *F*(1, 15) = 3.96, *p* = 0.065, showing a trend toward a larger dilation when S2 was the modulated stimulus, just as we observed in Experiment 1.

#### Bias

**Figure 3:**
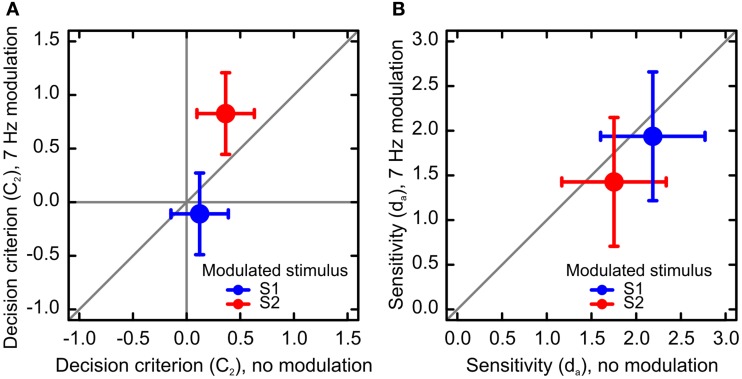
**Results of Experiment 2: bias and sensitivity**. **(A)** Results of the analysis of decision criterion. Negative values of the criterion indicate a bias toward S1, and positive values indicate a bias toward S2. When S1 was modulated, observers were more likely to report that S1 was the longest stimulus, and *vice versa* for S2. Criterion was also affected by stimulus order. Observers were likely to report that the second stimulus was longer than the first. **(B)** Results of the analysis of sensitivity. Sensitivity to duration was lower for modulated than non-modulated stimuli. Sensitivity also decreased when S2 was the standard stimulus, independent of stimulus modulation. The error bars represent 95% confidence intervals.

For each condition, we calculated criterion location C_2_: a measure of response bias that allows for unequal variances of the distributions of decision variables (Macmillan and Creelman, 2005). In a repeated measures analysis of criterion C_2_, using the same fixed effects as in the sensitivity analysis, and using categories of Observers and Response as random effects, we found that the criterion significantly depended on factors Modulation Frequency, *F*(1, 144) = 5.69, *p* < 0.05 and Standard Order, *F*(1, 144) = 55.06, *p* < 0.01. The two factors interacted, *F*(1, 144) = 19.47, *p* < 0.01, indicating that modulation induced a response bias for a longer duration of the modulated stimulus (Figure 3A).

#### Sensitivity

**Figure 4:**
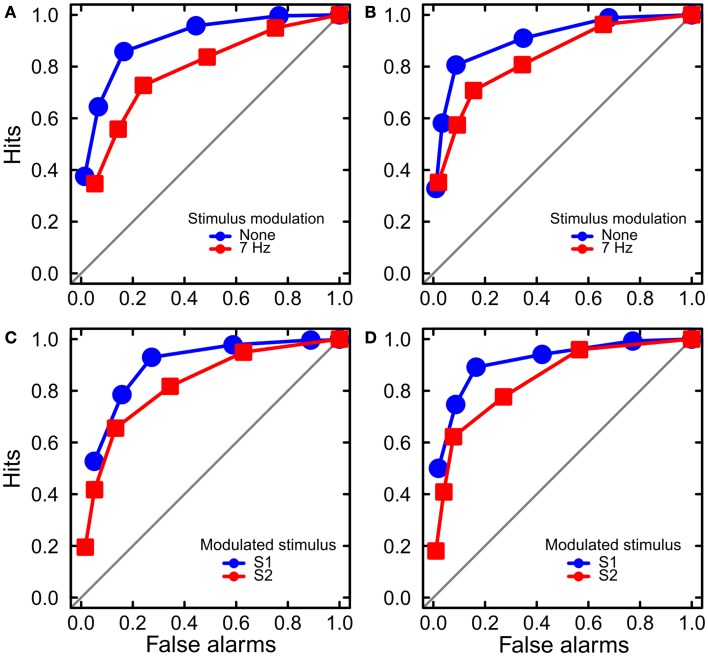
**Results of Experiment 2: ROC analysis**. The ROC curves are averaged across the six observers for two standard durations and two standard stimulus orders. **(A,B)** The curves are separated by the factor of stimulus modulation. Luminance modulation reduced sensitivity to stimulus duration. The data are shown separately for the standard durations of 500 ms **(A)** and 1100 ms **(B)**. **(C,D)** The curves are separated by the factor of stimulus order. Sensitivity was higher for the first (S1) than the second stimulus, for both standard durations of 500 ms **(C)** and 1100 ms **(D)**.

We obtained empirical ROC curves within every modulation frequency, stimulus order, standard duration, and observer by calculating hit and false-alarm rates for the six response categories (Figure 4). We then calculated sensitivity *d_a_*, which is a measure of sensitivity that allows for unequal variances in the probability distributions for stimuli S1 and S2 (Macmillan and Creelman, 2005). We then analyzed sensitivity using a mixed-effects model with factors Standard Duration (500 or 1100 ms), Modulation Frequency (0, 7 Hz), and Standard Order (S1, S2) as fixed effects, and Observer as a random effect. We found significant main effects for Modulation Frequency, *F*(1, 29) = 4.89, *p* < 0.05 and Standard Order, *F*(1, 29) = 10.36, *p* < 0.01 (Figure 3B). The two factors did not interact, *F*(1, 29) = 0.01.

To summarize, we replicated the time dilation effects of Experiment 1 using the method of constant stimuli. As in Experiment 1, dilation was larger for the modulated than non-modulated stimuli, and it was larger for the second stimulus (S2). In addition, we found that time dilation was associated with lower sensitivity to stimulus duration: sensitivity was lower for the modulated than non-modulated stimuli, and it was lower for S2 than S1. The decrements of sensitivity associated with stimulus modulation and stimulus order were additive, suggesting that the loss of sensitivity caused by luminance modulation was independent of the loss caused by stimulus order.

## Discussion

In two experiments we investigated how temporal articulation of visual stimuli affects their perceived duration. We created temporal articulation by making stimulus luminance a periodic function of time. Articulated stimuli appeared to have longer durations: the faster the luminance modulation (i.e., the higher its temporal frequency) the larger the apparent duration. An analysis of response ratings revealed that stimulus articulation had two effects: it biased the estimates toward longer durations, and it reduced observers’ sensitivity to duration.

Stimulus order also affected the perceived duration and the sensitivity to duration. The second stimulus of the sequence appeared to last longer than the first stimulus: the *time-order effect* (Fechner, 1860; Hellstrom, 1985). We found that sensitivity to the (dilated) second stimulus was lower than the sensitivity to the first stimulus. The respective losses of sensitivity due to luminance modulation and stimulus order were additive, suggesting that both were caused by separate processes.

Several explanations for time dilation have been proposed. According to the explanation from stimulus complexity (Fraisse, 1963, 1984; Ornstein, 1969), time dilation occurs because complex stimuli require more effort and storage space than simple stimuli. A similar explanation was advanced to account for effects of attention on duration estimation, suggesting that attention increases the information processing rate (Tse et al., 2004). However, these accounts do not explain why time dilation is associated with decreased sensitivity to duration.

One may argue that visual modulation attracts one’s attention and draws it away from the processing required for estimation of duration, thus reducing the ability to estimate stimulus offset and distorting the perceived duration. Below we propose that the attentional component of this view may be redundant because time dilation can be a consequence of a basic property of sensory measurement.

We explore the possibility that luminance modulation stimulates the visual mechanisms that are more suitable for measuring stimulus frequency content, at the expense of measuring other aspects of the stimulus, such as its duration. We illustrate how this process can affect duration estimation using numerical simulations.

### Uncertainty tradeoff

According to a basic constraint of information theory, called the *uncertainty principle* (Gabor, 1946), the uncertainties associated with simultaneous measurement of signal location and frequency content are not independent of one another (Resnikoff, 1989; Gepshtein et al., 2007). The product of these uncertainties is bounded by a constant, and so the uncertainty about temporal location of the signal cannot be reduced without increasing the uncertainty about its temporal frequency content, and *vice versa*. As a result, the mechanisms optimized for estimation of frequency content are expected to integrate stimulation over longer temporal intervals. A side effect of this optimization is an increased uncertainty about the temporal location of the signal.

Applying this principle to sensory estimation of stimulus duration, we expect that outcomes of estimation will behave in a manner consistent with the tradeoff of uncertainties. That is, we expect that increasing the salience of stimulus frequency content will engage visual mechanisms specialized for measurement of stimulus frequency content which, by the nature of this specialization, integrate visual stimuli over longer temporal intervals than mechanisms specialized for other tasks. Time dilation and reduced sensitivity to stimulus duration are immediate consequences of measurement with larger temporal intervals, as we show next.

### Numerical illustration

**Figure 5:**
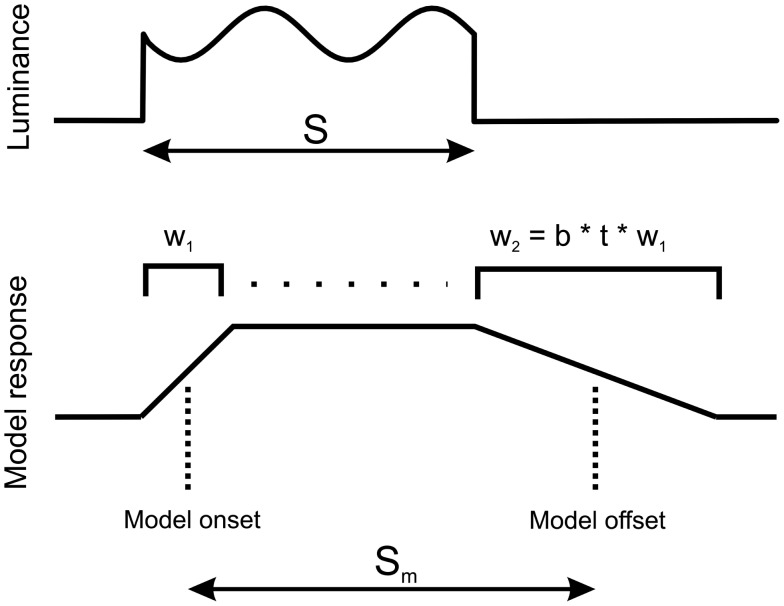
**Model of duration measurement**. Luminance of the stimulus (top) is integrated using a window whose duration *w_i_* is a function of time and stimulus modulation. At stimulus onset, window length is *w_1_*, which then increases toward length *w_2_* at stimulus offset (as explained in the main text). The onset and offset of model response are estimated at the half of the maximal value of model response (bottom). *S* and *S_m_* are the durations of the stimulus and model response, respectively. Since stimulus offset is estimated using a longer window than stimulus onset, response to stimulus offset is delayed, causing an apparent increase of stimulus duration.

Let the perceived duration of a sensory event be the time elapsed between the estimated onset and offset of the event. Let the sensory input be integrated over temporal windows of different durations, represented by *measurement window* *W* (Figure 5). The estimated stimulus onset and offset are defined as the moments at which the outcomes of integration reach some threshold, here modeled as half of the maximal magnitude of the outcome of integration.

From the above argument of uncertainty tradeoff, we expect that the presence of luminance modulation in the stimulus causes an increase of the length of temporal integration. Hence, the offsets of modulated stimuli ought to be integrated using longer intervals than the onsets (The respective intervals – or integration “windows” – are labeled in Figure 5 as *w_2_* and *w_1_*.). The elongation of integration windows results in a delayed detection of stimulus offset, and thus in time dilation. For simplicity, let the length of the integration interval be a linear function of the time elapsed since stimulus onset, such that interval length grows at a rate that depends on the stimulus content:

$$w_{2}=b\;t\;w_{1},$$

where *t* > 0 is the elapsed time, and *b* > 0 depends on the stimulus content.

To model the comparison of durations of two successive stimuli, we implement an assumption common in models of temporal estimation, that the estimated duration is a decaying function of time (Köhler, 1923). In agreement with results of Experiment 2, where we found that the changes of sensitivity due to stimulus modulation and due to stimulus order were independent of one another, we assume that the effect of stimulus modulation is separate from the effect of stimulus order.

**Figure 6:**
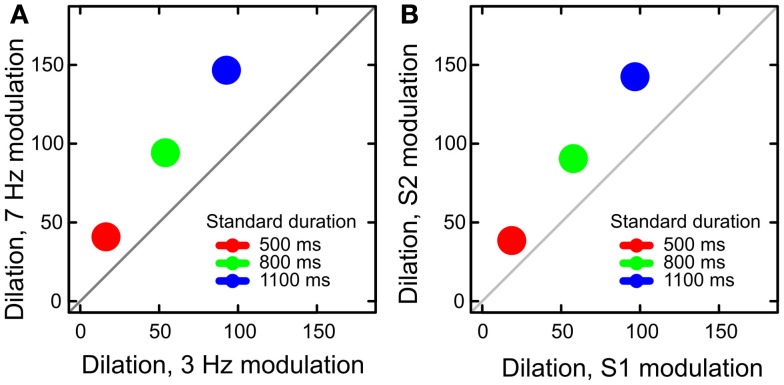
**Results of numerical simulation**. **(A)** The amount of dilation predicted by the model is increased by stimulus modulation and it grows with stimulus duration. **(B)** Dilation was larger when the second stimulus (S2) was modulated than when S1 was modulated. This effect increased with stimulus duration.

This simple model reproduces the key results of Experiments 1 and 2. Model predictions are summarized in Figure 6: for three standard stimulus durations, two levels of luminance modulation, and two orderings of the modulated stimulus, assuming a fixed ISI. The model predicts that time dilation depends on the amount of luminance modulation (Figure 6A) and that dilation increases with increased stimulus duration (Figure 6B). The model also predicts an interaction between stimulus duration and modulation order (Figure 6B), as we found in Experiment 1, and an interaction between stimulus duration and the amount of modulation (Figure 6A), similar to the trend observed in Experiment 1. Moreover, the simulated dilation increases when the second stimulus (S2) is modulated, and this increase is larger for the longer stimulus durations.

To summarize, the simulations have shown that perceptual consequences of a basic principle of measurement can explain both time dilation and the reduced sensitivity associated with luminance modulation of visual stimuli. Time dilation was previously found to increase with modulation frequency (as in our Experiment 1) up to the frequency of 7 Hz, after which this trend saturated (Kanai et al., 2006). In our framework, the saturation of time dilation is expected because visual sensitivity to temporal modulations of luminance peaks between the frequencies of 5 and 10 Hz (Kelly, 1979). Luminance modulation with frequencies within this interval will therefore be most effective in recruiting long intervals of integration, resulting in a maximal effect on the estimation of duration.

In the numerical simulations, we assumed a distinct mechanism engaged in estimating the time elapsed between stimulus onsets and offsets. Such “time tracking” mechanisms are not well understood, sometimes described as clocks (Eagleman et al., 2005; Ivry and Schlerf, 2008), as pulse counting mechanisms (reviewed in Allan, 1979), or as distributed neural systems (Mauk and Buonomano, 2004). We made no assumptions specific to any of these hypotheses. That is, we propose that time dilation can be explained independently of the specific mechanism used for time tracking.

The effects predicted by our model are similar to the effects of attention on temporal processing. For example, when two flashes appeared in a cued location, observers saw them as one flash more often than when the flashes appeared in an unattended location (Yeshurun and Levy, 2003), as if attention increased the duration of the measurement interval. Similarly, when a cue correctly identified the location of a subsequent stimulus, the perceived duration of the stimulus was longer than in invalidly cued locations (Mattes and Ulrich, 1998). It is plausible that, to reduce uncertainty about the content of attended stimuli, cueing engages mechanisms characterized by longer temporal intervals. Integration of stimuli over such longer intervals will delay the apparent instant of signal offset and impair the ability to distinguish sequential events.

The notion that attention extends the duration of temporal integration (and thus delays the detection of stimulus offset) has been supported by studies of reaction times. For example, in the study of Rolke et al. (2006), observers responded to onsets or offsets of stimuli preceded by valid or neutral cues about stimulus spatial location. Reaction times to offsets of the cued stimuli lagged, as compared to reaction times to the neutrally cued stimuli. These results show that attention to spatial location may cause loss of sensitivity to temporal location, indicating that spatial and temporal parameters of stimuli are not measured independently of one another.

The evidence of association between spatial and temporal extents of measurement brings to mind some well-known characteristics of visual mechanisms. For example, populations of parvocellular and magnocellular cells are characterized by such spatiotemporal tradeoffs. Parvocellular cells are characterized by small receptive fields that cover the central visual field and have a low temporal resolution, whereas magnocellular cells are characterized by larger receptive fields that cover retinal periphery and have a higher temporal resolution. Effects of attention on temporal integration in different parts of the visual field have been attributed to such selective activation of parvocellular and magnocellular cells (Yeshurun and Levy, 2003; Rolke et al., 2006).

Further evidence for the association of spatial and temporal extents of measurement is found in neuroimaging studies. Different durations of temporal integration intervals are distributed hierarchically across areas of the visual cortex: mechanisms residing in the “early” visual areas are characterized by shorter temporal intervals than in the “higher-level” areas, where properties of objects are represented (e.g., Hasson et al., 2008). In view of our proposal about the role of integration intervals for perception of duration, these findings suggest an explanation for the previous finding that events involving complex objects appear to have longer durations than simple objects (Goldfarb and Goldstone, 1963; Schiffman and Bobko, 1974; Allan, 1979; Fraisse, 1984). Complex objects appear to have longer durations than simple objects because perception of complex objects depends on mechanisms characterized by long temporal intervals of measurement, at the cost of lower temporal resolution.

In summary, we found that time dilation is accompanied by decreased sensitivity to duration. We have shown that the decreased sensitivity is consistent with the notion that length of temporal integration varies according to the temporal frequency content of visual stimulus.

## Conflict of Interest Statement

The authors declare that the research was conducted in the absence of any commercial or financial relationships that could be construed as a potential conflict of interest.
